# Research on an Electromagnetic Compatibility Test Method for Connected Automotive Communication Antennas

**DOI:** 10.3390/s25061922

**Published:** 2025-03-19

**Authors:** Ming Ye, Wenxia Wang, Jin Jia, Weixin Cai, Qi Cao, Kai Sheng

**Affiliations:** 1 Key Laboratory of Advanced Manufacturing Technology for Automobile Parts, Ministry of Education, Chongqing University of Technology, Chongqing 400054, China; cqyeming@cqut.edu.cn (M.Y.); 1119372252@stu.cqut.edu.cn (W.W.); cwxllxx@stu.cqut.edu.cn (W.C.); shengkai@stu.cqut.edu.cn (K.S.); 2 Army Logistics University, Chongqing 401331, China; roy1976@163.com

**Keywords:** automotive OTA antenna, antenna gain coupling, antenna radiation simulation, electromagnetic compatibility

## Abstract

Based on the problem of electromagnetic interference in the darkroom of connected vehicle communication systems, a research method is proposed to evaluate the wireless communication quality between antennas using gain coupling. The electromagnetic compatibility of the far-field radiation pattern, radiation coupling degree, and near-field radiation pattern of the vehicle antenna and the vehicle antenna model were analyzed by establishing a 3D simulation model of the vehicle antenna and the external transmitting antenna. Based on the obtained simulation data, the arrangement scheme of the transmitting antenna in the darkroom can be determined. The performance index of the automotive OTA antenna can then be tested using the determined arrangement scheme, which can effectively reduce costs and provide reliable data.

## 1. Introduction

As the number of automotive antennas and related wireless protocols increases, the functionality of modern vehicles’ communication subsystems resembles that of traditional mobile user devices. Therefore, there is a growing demand for the expansion of automotive antenna measurement systems, leading to the need for mature OTA testing facilities. These testing systems typically require SNF data for a hemisphere surrounding the vehicle. The vehicle is placed on a turntable and rotated, while a rotating gantry or a fixed arch with a movable probe or probe array collects amplitude and phase data and converts them into far-field measurements. The large electrical size of these vehicles, coupled with the lack of formal OTA standards in the automotive industry, makes the design of such systems quite challenging [1,2].

Currently, common testing methods include multiprobe [3] and two-stage testing methods [4]. In the multiprobe testing method, multiple downlink signals output from a base station simulator first enter a channel emulator during the testing process. The channel emulator applies fading to the downlink signals from the base station simulator according to the channel scenario configured in the system, calculating the incident angles, power delays, angular spreads, and polarization directions of the multipath downlink signals around the terminal after fading based on the specified channel model. Signals with different incident angles are then mapped to the corresponding probes in the anechoic chamber. Multiple probes in the anechoic chamber transmit simultaneously based on the loaded results from the channel emulator, thus simulating downlink signals with different arrival angles, powers, polarizations, and delay characteristics in the testing area. Generally, testing systems require the configuration of multichannel amplifiers to amplify the downlink signals reaching the multiple probes, ensuring that the downlink power in the testing area maintains a stable and reliable connection. The first phase of the two-stage method is to obtain the antenna radiation pattern; the near-field radiation pattern of the entire vehicle antenna is measured in an anechoic chamber and transformed into the far-field radiation pattern, representing the radiation pattern of the DUT. The second phase involves loading the radiation pattern information measured in the first phase into the channel emulator to simulate a wireless channel that includes the characteristics of the DUT antenna. The downlink signals output by the base station simulator are convolved with the wireless channel that has the DUT direction pattern information loaded and then emitted via the measurement antenna for receiver performance testing.

On 12 June 2024, the Global Automotive Information Communication Evaluation Technology Innovation Base, funded by China Automotive Research in Chongqing with a total investment of nearly 350 million yuan, implemented a testing method to evaluate the performance of wireless communication terminal antennas. This method simulated the transmission scenario of product wireless signals in the air, comprehensively validating the impact of various factors on overall performance from the chip to the antenna end. The laboratory adopted the most widely accepted whole-vehicle spherical near-field testing scheme in the industry, meeting testing requirements for passive and active performance of antennas related to cellular communication, positioning, short-range communication, and C-V2X. This also established industry-leading capabilities for automotive communication research and development testing services [5].

Currently, automotive communication testing continues to rely on methods utilizing small-scale electrical components. Traditional testing approaches are not only complex in their procedure, but also often entail substantial cost investments. These high expenses and cumbersome testing processes pose significant challenges to research endeavors and considerably impede the rapid advancement of practical engineering applications. This study proposes the utilization of electromagnetic compatibility characteristics to conduct antenna communication testing.

If important sampling points in the anechoic chamber can be determined through preliminary simulations, testing costs and complexity can be significantly reduced. This paper proposes an electromagnetic compatibility testing method for communication antennas under actual working conditions. The first step is to select the antenna model and simulate the gain radiation coupling between antennas through their far-field radiation pattern, comparing it with radiation values from relevant standards to verify feasibility. By indicating the levels of coupling gain values between the radiation patterns, we can demonstrate the extent of interaction between these two antennas, leading to conclusions about the strength of wireless communication capabilities between antennas. This allows for precise debugging and optimization of S-parameters through simulation software, thereby enhancing the stability of communication systems and achieving cost reduction, along with repeatability and comparability of testing results.

Currently, communication testing in automobiles still relies on methods designed for small-scale electrical components. When these methods are applied to vehicles, the testing process becomes considerably more complex and often requires substantial financial investment. The high costs and cumbersome procedures not only pose significant challenges to research endeavors, but also substantially impede the rapid advancement of practical engineering applications. The testing method proposed in this study ensures the integrity and reliability of electromagnetic signal transmission at the very first step—signal reception—while also enabling long-distance propagation. Compared to traditional testing methods, the approach presented in this study simplifies the testing process and reduces associated costs.

## 2. Electromagnetic Radiation Testing Method

Electromagnetic compatibility and communication antennas are two important fields in modern electronic and communication systems. They are interconnected, ensuring the normal operation of devices and good communication quality. The electromagnetic compatibility of communication equipment significantly affects communication quality and the stability of communication networks. In the field of wireless communication, due to the limited frequency resources, different communication systems must coexist within the same frequency bands. By utilizing the gain from antennas during reception and transmission to arrange testing environments, we can attempt to replicate the working state of communication antennas in complex real-world environments, improving overall system performance and reliability. In this regard, electromagnetic compatibility testing has become a necessary step in the development of communication equipment.

### 2.1. Determining the Test Antenna Type and Model

This paper primarily conducted an in-depth study of communication capabilities through antenna gain and coupling effects, making antenna performance particularly important. Specifically, the antenna’s gain and its omnidirectional radiation pattern are the main reference standards in the simulation testing system. To discuss antennas communicating with the base station, 4G antennas on vehicles are typically selected, while GNSS antennas on vehicles are used for satellite communication.

**Figure 1:**
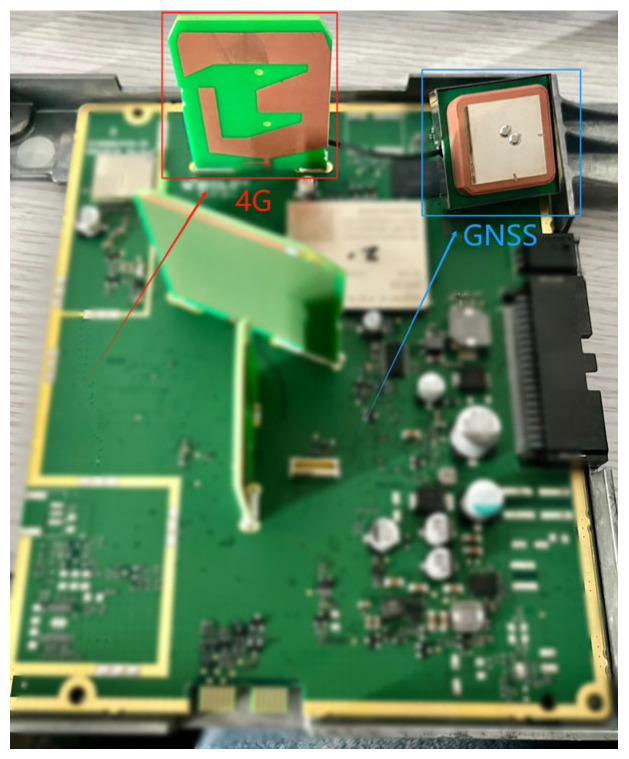
GNSS and 4G antennas in the shark fin.

In this study, we selected a shark-fin antenna from a certain brand vehicle as the antenna for testing based on the project requirements. A shark-fin antenna integrates multiple antennas, and while various forms of antennas are deployed on vehicles today, the methodology of this study is grounded in electromagnetic compatibility testing. Consequently, this approach is applicable regardless of the type or configuration of the antenna. Electromagnetic compatibility testing not only serves to assess communication with transmitting antennas, but also utilizes electromagnetic compatibility characteristics to mitigate interference among vehicle-mounted antennas. This is illustrated in Figure 1. The shark-fin antenna, notable for its distinctive design and superior gain characteristics, has garnered significant attention for its ability to enhance signal transmission capabilities in complex communication environments.

**Figure 2:**
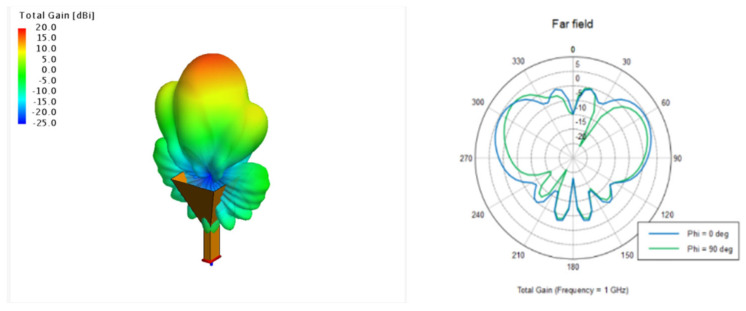
The antenna radiation patterns representing the base station and satellite transmitting antennas.

Based on the antenna pattern characteristics of the 4G and GNSS antennas in the antenna library of the electromagnetic software FEKO 2023, we selected the base station transmission and satellite transmission antennas. The directional pattern characteristics and antenna selections are shown in Figure 2.

One of the most basic methods during the automotive antenna testing process is passive testing. However, this testing method has the drawback of only evaluating the antenna’s performance and not providing a comprehensive functional validation. Therefore, to achieve a more accurate and comprehensive assessment, we employed the electromagnetic radiation testing method, which is based on the overall testing scheme of the complete vehicle. By installing the antenna on the entire vehicle, we treated the antenna and the vehicle as a whole to solve the near- and far-field radiation patterns.

**Figure 3:**
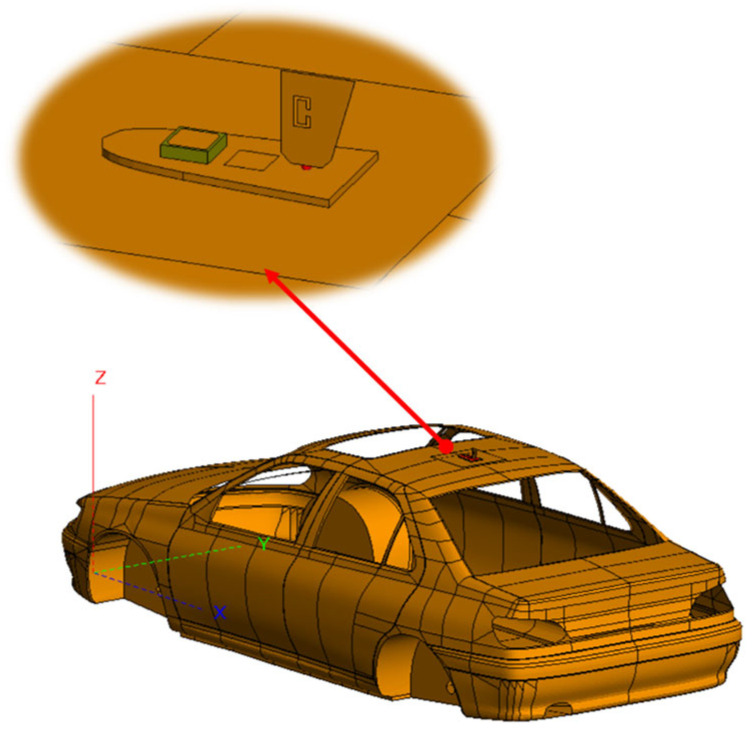
Schematic diagram of the vehicle—mounted antenna arrangement.

After establishing the model, we further designed and analyzed the layout of the 4G antenna, the GNSS antenna, and the vehicle model, as detailed in Figure 3.

Due to the size limitations of vehicles, far-field testing faces numerous challenges during implementation. With the increasing application of complex antennas, traditional far-field testing methods have struggled to meet the requirements of testing tasks. Therefore, near-field testing has gradually become a relatively reliable and effective measurement approach. After performing near-field testing, we typically apply the near-to-far-field transformation theory and relevant algorithm formulas to convert near-field data into far-field models. The formula employed in this study was established using the FEKO software. Since it has been validated by FEKO, the derived formula is directly referenced in this research [6].

(1) $$\begin{array}{c} E_{\theta }(r,\theta ,\pi )=\sum_{n=0}^{N}\sum_{m=-n}^{n}C_{mn}e^{jm\varphi }\left\{a_{mn}\frac{jmP_{n}^{\left|m\right|}(\cos\theta )}{\sin\theta }H_{n}^{\left(2\right)}(kr)\right.\left.+b_{mn}\frac{dP_{n}^{\left|m\right|}(\cos\theta )}{d\theta }g_{n}(kr)\right\} \end{array}$$

(2) $$\begin{array}{c} E_{\varphi }(r,\theta ,\pi )=\sum_{n=0}^{N}\sum_{m=-n}^{n}C_{mn}e^{jm\varphi }\left\{-a_{mn}\frac{dP_{n}^{\left|m\right|}(\cos\theta )}{d\theta }H_{n}^{\left(2\right)}(kr)\right.\left.+b_{mn}\frac{jmP_{n}^{\left|m\right|}(\cos\theta )}{\sin\theta }g_{n}(kr)\right\} \end{array}$$

When r→+∞, $H_{n}^{\left(2\right)}(kr)\approx \frac{j^{n+1}e^{-jkr}}{kr}$, and $g_{n}(kr)\approx \frac{j^{n}e^{-jkr}}{kr}$, factors unrelated to θ,φ can be omitted to derive the electric field in the far-field region of the antenna:

(3) $$\begin{array}{c} E_{\theta }(\theta ,\pi )=\sum_{n=0}^{N}\sum_{m=-n}^{n}C_{mn}e^{jm\varphi }j^{n}\left\{-a_{mn}\frac{mP_{n}^{\left|m\right|}(\cos\theta )}{\sin\theta }\right.\left.+b_{mn}\frac{dP_{n}^{\left|m\right|}(\cos\theta )}{d\theta }\right\} \end{array}$$

(4) $$\begin{array}{c} E_{\varphi }(\theta ,\pi )=\sum_{n=0}^{N}\sum_{m=-n}^{n}C_{mn}e^{jm\varphi }j^{n+1}\left\{-a_{mn}\frac{dP_{n}^{\left|m\right|}(\cos\theta )}{d\theta }\right.\left.+b_{mn}\frac{mP_{n}^{\left|m\right|}(\cos\theta )}{\sin\theta }\right\} \end{array}$$

(5) $$\begin{array}{c} a_{mn}=\frac{-1}{H_{n}^{\left(2\right)}(kR_{0})}\left\{\int_{0}^{\pi }\left[\int_{0}^{2\pi }V_{\theta }(R_{0},\theta^{\prime },\varphi^{\prime })e^{-jm\varphi^{\prime }}d\varphi^{\prime }\right]\frac{jmC_{mn}P_{n}^{\left|m\right|}(\cos\theta^{\prime })}{\sin\theta^{\prime }}\sin\theta^{\prime }d\theta^{\prime }\right.\\ \left.+\int_{0}^{\pi }\left[\int_{0}^{2\pi }V_{\varphi }(R_{0},\theta^{\prime },\varphi^{\prime })e^{-jm\varphi^{\prime }}d\varphi^{\prime }\right]\frac{C_{mn}dP_{n}^{\left|m\right|}(\cos\theta^{\prime })}{d\theta^{\prime }}\sin\theta^{\prime }d\theta^{\prime }\right\} \end{array}$$

(6) $$\begin{array}{c} b_{mn}=\frac{1}{g_{n}(kR_{0})}\left\{\int_{0}^{\pi }\left[\int_{0}^{2\pi }V_{\theta }(R_{0},\theta^{\prime },\varphi^{\prime })e^{-jm\varphi^{\prime }}d\varphi^{\prime }\right]\frac{C_{mn}P_{n}^{\left|m\right|}(\cos\theta^{\prime })}{\sin\theta^{\prime }}\sin\theta^{\prime }d\theta^{\prime }\right.\\ \left.-\int_{0}^{\pi }\left[\int_{0}^{2\pi }V_{\varphi }(R_{0},\theta^{\prime },\varphi^{\prime })e^{-jm\varphi^{\prime }}d\varphi^{\prime }\right]\frac{jmC_{mn}dP_{n}^{\left|m\right|}(\cos\theta^{\prime })}{d\theta^{\prime }}\sin\theta^{\prime }d\theta^{\prime }\right\} \end{array}$$

where the radius of the smallest sphere surrounding the tested antenna is $r_{\min}$, and r within the passive zone is $r\geq r_{\min}$; the tangential electric field components at the near-field distance from the coordinate origin $R_{0}$ are $V_{\theta }(R_{0},\theta^{\prime },\varphi^{\prime }),\;V_{\varphi }(R_{0},\theta^{\prime },\varphi^{\prime }),\;E(r,\theta ,\varphi )$, representing the vector sets of the spherical wave in three directions; $H_{n}^{\left(2\right)}(kr)$ indicates the spherical Hankel function; $P_{n}^{\left|m\right|}(\cos\theta )$ represents the associated Legendre function; $a_{mn},\;b_{mn}$, and the respective coefficients $M_{mn},\;N_{mn}$ are complex amplitude coefficients, containing both far- and near-field information; N is the order of the highest-order mode in the antenna expansion formula, $N\approx kr_{\min}+10,N$, taking integers; and k is the wavenumber, k=2π/λ.

**Figure 4:**
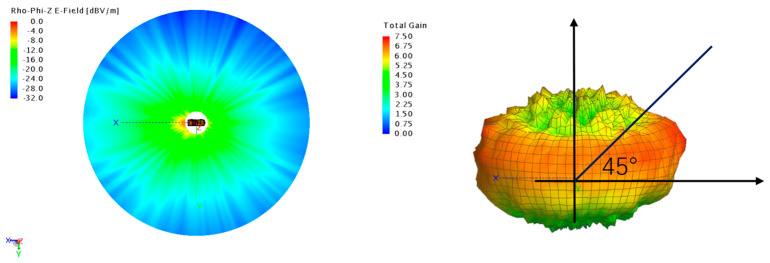
Near—and far—field radiation patterns of the vehicle—level antenna system.

Based on the near-to-far-field algorithm formulas, we obtained the far-field radiation pattern, as shown in Figure 4. By analyzing the omnidirectional far-field radiation pattern, we can clearly see that the gain intensity of the vehicle antenna primarily concentrates in the direction forming a 45-degree angle with the horizontal plane. This finding provides an important theoretical basis for the subsequent selection of the base station and satellite placement in the anechoic chamber. Specifically, the distribution characteristics of the antenna gain in this particular angular range indicate that positioning the base station and satellite in the optimal direction of the vehicle antenna gain can significantly enhance signal transmission efficiency and quality. Thus, this research outcome not only aids in optimizing the antenna layout, but also lays the foundation for improving the performance of the entire wireless communication network.

### 2.2. Analyzing Connected Vehicle Conditions in Urban Environments

When evaluating wireless terminal communication performance in real scenarios, one effective method is road testing technology, which involves manually conducting actual communication performance evaluations in high-rise buildings or suburban vegetation areas. In practice, road testing technology has shown limitations in meeting all the wireless measurement requirements, mainly due to the following characteristics. First, the electromagnetic radiation in the surrounding environment during road tests is unknown and uncontrollable, such as during rainy or sunny days when the electromagnetic parameters of the propagation environment fluctuate; thus, measurement results often lack high repeatability and comparability. Second, the cost and time of measurements are high, with road test evaluations often requiring several days or even weeks of repeated measurements to arrive at converging conclusions.

**Figure 5:**
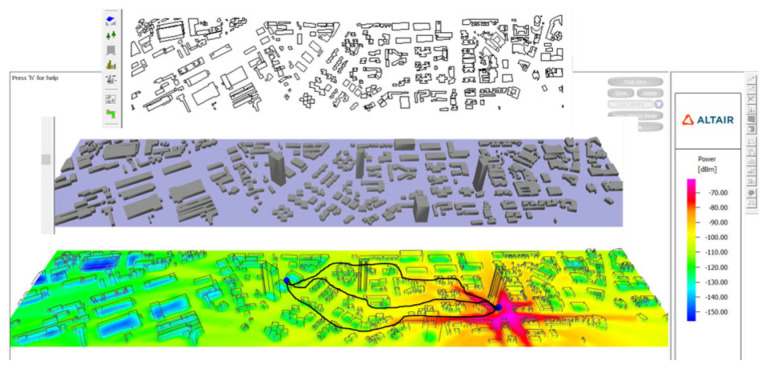
Simulation of the radiation path from the base station to the vehicle terminal in an urban environment.

First, we established a two-dimensional model of the city using WinProp 2023 software, which we then converted into a three-dimensional visual model [7]. To achieve a more accurate simulation, we placed an omnidirectional antenna on the rooftop of a high-rise building to simulate the base station’s functionality while simulating a 4G receiving antenna in a car on another street. This setup allowed us to comprehensively analyze and assess the wireless signals propagated by the base station. The simulation results displayed the omnidirectional radiation gain status of the base station relative to the vehicle, as depicted in Figure 5. Further observation of its radiation path revealed that the radiation path from the base station was essentially consistent with the signal propagation path to the antenna. This finding enabled us to confirm the signal propagation characteristics between the base station and the vehicle, allowing us to analyze antenna radiation properties to more accurately describe the signal path.

**Figure 6:**
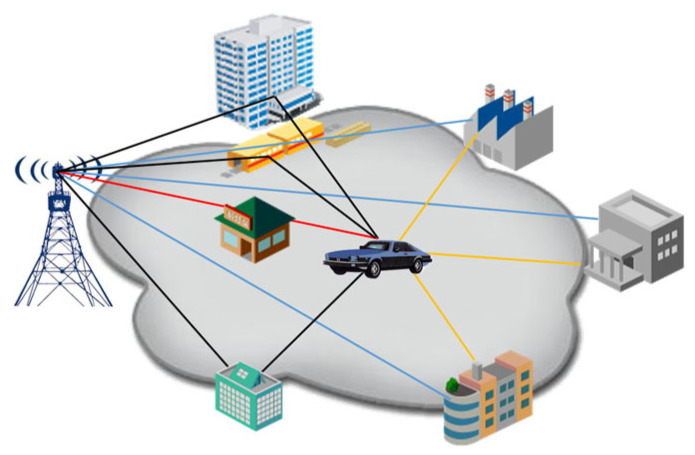
The multipath effects of connected vehicles in urban environments.

Further analysis of channel transmission conditions for connected vehicles in urban environments revealed that the vehicle, while in motion, receives signals transmitted from different directions. As the complexity of the surrounding environment increases, multipath effects become more pronounced, and the number of NLOS paths also increases, as illustrated in Figure 6. These environmental factors significantly influence signal propagation; therefore, special attention must be paid to spatial correlation coefficients when designing an antenna communication testing system for the complete vehicle.

Through this analysis, we recognize that onboard antennas in urban environments are influenced by multiple factors. These factors specifically include the angle distribution of multipath effects, the spacing between antennas, the coupling characteristics of antenna gain, and the radiation directions. The combined effects of these factors not only affect the quality and stability of the signal, but also place higher demands on the vehicle’s communication performance in dynamic environments. Thus, considering the interactions of these parameters will be crucial in the design and layout of connected vehicle antenna systems.

### 2.3. Constructing a Three-Dimensional Electromagnetic Model Environment

**Figure 7:**
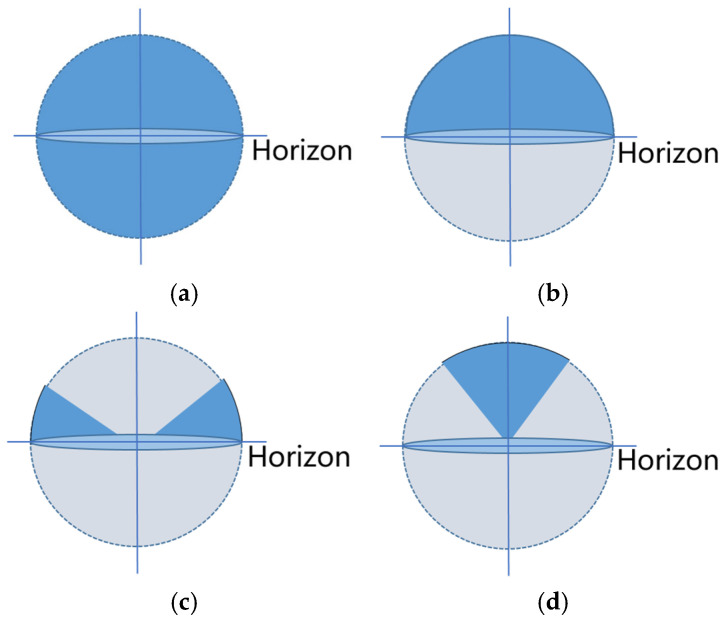
The communication range of interest for connected vehicles corresponds to the deep blue region ((**a**) test area based on small-sized electrical equipment; (**b**) test area based above the horizontal plane; (**c**) test area suitable for base station transmitting antennas; (**d**) test area suitable for satellite transmitting antennas).

The MIMO scene configuration for small-sized electrical devices is depicted in Figure 7a, with the deep blue area covering the complete sphere of interest. The key performance indicators for this configuration include return loss, throughput, and delay. Although this encompassing approach is quite comprehensive, its measurement methods may not effectively evaluate antenna performance on vehicles due to the unique characteristics of ground-based travel.

Thus, Figure 7b focuses specifically on the configuration of the upper hemisphere, with key indicators including return loss, throughput, and delay across the upper hemisphere. This definition of the upper hemisphere can, to some extent, be more effective in assessing the overall performance of the onboard satellite communication system. By concentrating on the signal propagation characteristics of the upper hemisphere, we can better understand antenna performance under actual working conditions.

Based on the gain characteristics of the far-field radiation pattern of the complete vehicle and the complex radiation paths experienced by connected vehicles in urban environments, we can further segment the areas of interest. Figure 7c depicts a specific spherical area around the horizon. This area is particularly sensitive to base station, V2V, and V2I communication, with performance indicators including near-ground return loss, throughput, and delay. Figure 7d demonstrates the spherical area of interest surrounding the zenith. This area is especially sensitive to signal performance for GNSS and satellite communications. The performance indicators for this area include return loss, throughput, and delay for the near-zenith portion, which are crucial for ensuring the quality of satellite communication.

Through in-depth analysis of these two areas of interest, we can comprehensively understand the communication needs of connected vehicles in complex urban environments. This segmentation aids in achieving environmental design, improving signal transmission reliability, and enhancing communication efficiency and safety between vehicles and base stations, as well as the GNSS equipment. Furthermore, an analysis of these key indicators showed that both the base station and the GNSS areas include throughput as a performance metric; therefore, throughput was chosen as the reference indicator for antenna performance in the electromagnetic radiation testing method.

**Figure 8:**
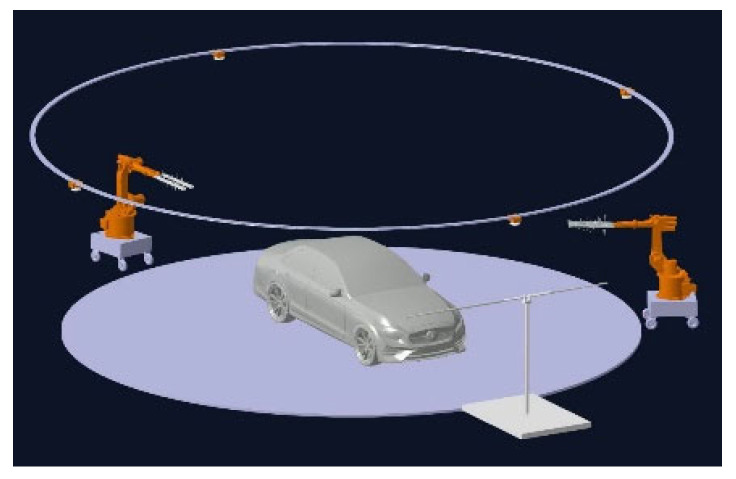
Three-dimensional schematic diagram of the connected vehicle testing model.

After comprehensive analysis and research of the actual conditions, we applied the three-dimensional modeling software CATIA P3 V5R21to systematically arrange the configurations in the anechoic chamber, as shown in Figure 8. This layout can generally be divided into three main components. First, the whole vehicle model acts as a communication antenna receiver, responsible for receiving communication information from various signal sources. Second, the base station transmitting antenna is controlled by a robotic arm to adjust its transmission angle, allowing it to move flexibly within the space above the blue area in Figure 7c. This dynamic control ensures optimal signal coverage, enhancing the stability and reliability of communications. Lastly, the GNSS transmitting antenna is arranged at the edge of the blue area in Figure 7d, simulating the trajectory of the satellite’s launch path. This design not only accurately simulates the actual emission of satellite signals, but also aids in assessing the performance of onboard equipment in real-time navigation and positioning.

**Figure 9:**
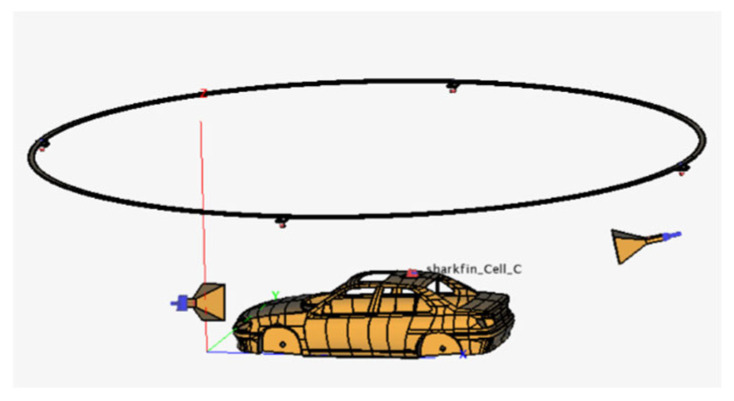
The three-dimensional electromagnetic simulation model.

This comprehensive arrangement method ensures coordination and cooperation among various modules, making the entire testing process more efficient and reliable. Finally, based on the three-dimensional model, we replicated the environment in electromagnetic software, as shown in Figure 9. Since we already analyzed and determined the antenna to be used in Chapter 1, during the model reconstruction process, the model was directly imported from the antenna library. When importing the model, it was necessary to set the solver type of each model to “MLFMM” to ensure consistency. However, mesh generation is a crucial step, as it directly affects the accuracy of the simulation and the consumption of computational resources. After importing the model, “Automatic meshing” should be clicked, and the system automatically generates the mesh for the model. If errors such as problematic triangles or other mesh generation issues occur during runtime, it is necessary to identify the specific erroneous surfaces in POSTFEKO and make local modifications accordingly. Another important point to note is that all models must use “Media” of “Perfect Electric Conductor” and “Perfect Magnetic Conductor”. The selected base station transmitting antenna, the GNSS transmitting antenna, and the vehicle-mounted receiving antenna should be positioned at their respective locations. After all the models are imported and properly positioned, it is essential to select all models and perform a Boolean operation, specifically the “Union” function in the toolbar. This step is of significant importance. Subsequently, the frequency required for the simulation should be set. Based on the frequency ranges of 4G and GNSS, the frequency range is ultimately set between 1 and 1.6 GHz. Finally, the solution type should be configured, typically selecting “Voltage Source”. Under “Configuration Specific”, “Far Fields” and “Near Fields” should be chosen from the “Requests” section, as these are the solution results relevant to this study. At this point, the setup of the simulation software is essentially complete. It is worth noting that the proposed method in this study requires testing in a fully anechoic chamber. Therefore, when conducting simulations in the electromagnetic simulation software FEKO, the default setting assumes an environment equivalent to a fully anechoic chamber. However, if conditions such as ground reflections need to be incorporated, these can be manually configured within the software to suit specific requirements.

Next, the coupling coefficient S-parameter values between the transmitting antenna and the vehicle-mounted receiving antenna were simulated.

## 3. Principles of the Electromagnetic Radiation Testing Method

Through in-depth analysis of existing standards, we can explore the interrelationship between S-parameters and throughput. This analysis can not only help identify the potential connection between the two, but also provide critical evidence for assessing communication quality in connected vehicles. S-parameters, or scattering parameters, are key parameters describing the signal reflection and transmission characteristics between RF and microwave components, while throughput refers to the amount of data successfully transmitted per unit of time by the system. By studying the relationship between S-parameters and throughput in depth, we can effectively evaluate the communication quality of connected vehicles and provide scientific evidence for improving their application effectiveness. This process can lay a theoretical foundation for advancing intelligent transportation and connected vehicle technologies, as well as guide the establishment of industry standards.

### Principle Analysis Based on the Electromagnetic Radiation Testing Method

In the design and operation process of wireless communication systems for connected vehicles, the MCL is a key parameter closely related to the definitions and calculations of S-parameters. Hence, when designing wireless communication systems for connected vehicles, the influence of S-parameters on the MCL must be comprehensively considered. By reasonably optimizing S-parameters, coupling losses can be effectively controlled, thus enhancing the coverage and service quality of the system. Moreover, understanding and applying these parameters is vital for improving the efficiency of wireless communication systems in connected vehicles, providing important reference for future development.

The MCL refers to the total channel loss mobile UE experiences between its antenna port and the base station. In calculating the MCL, the antenna’s directional gain is not considered, allowing the MCL to provide a simplified metric for assessing the quality of communication links. Generally, a higher MCL value indicates a stronger signal link, enhancing the system’s anti-interference capability and robustness.

The calculation formula for the MCL is as follows: uplink MCL = uplink maximum transmit power—base station receive sensitivity (NF + SINR + thermal noise); downlink MCL = downlink maximum transmit power—terminal receive sensitivity (NF + SINR + thermal noise). Since we only studied the communication sent by the base station and satellites to connected vehicles, we only utilized the calculation formula for the downlink MCL [8].

Assuming the external environment of the receiving terminal remains unchanged, the terminal’s receive sensitivity can be considered a fixed value. This implies that if the receiver sensitivity does not change, then there exists a proportional relationship between the downlink power and the downlink MCL. This relationship suggests that increasing the downlink power can effectively improve the signal quality, thereby enhancing communication reliability.

Once the downlink power and MCL exhibit a proportional relationship, we can leverage the correlation between the downlink power and throughput to indirectly deduce the mathematical relationship between the MCL and throughput. This correlation implies that as the downlink power increases, the throughput may also improve, suggesting that variations in the MCL could significantly impact throughput. Specifically, if we continually increase the downlink power while keeping the other conditions constant, an increase in the MCL could lead to improved signal quality, thus influencing the final throughput. Therefore, the MCL not only serves as a characterization index of link quality, but also has a direct impact on the transmission capacity of connected vehicle communication systems.

Through this investigation of indirect relationships, we can adjust wireless network parameters more effectively, enhancing the quality of wireless communication services.

**Figure 10:**
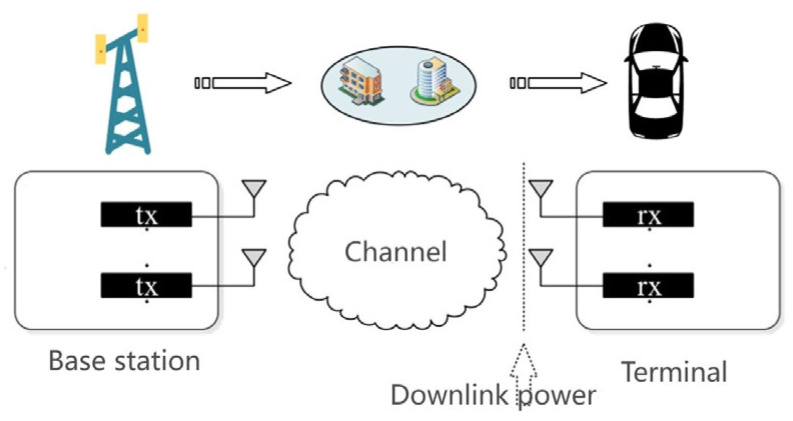
MIMO wireless communication schematic and definition of downlink power.

According to standards [9,10], the objective of throughput measurement is to establish a relationship between wireless terminal throughput and downlink power. The MIMO wireless communication schematic and the definition of downlink power are illustrated in Figure 10, where the signal transmitted by the base station reaches the terminal antenna front through a channel and is coupled into the receiver, defining the strength of the signal reaching the terminal antenna front as downlink power.

**Figure 11:**
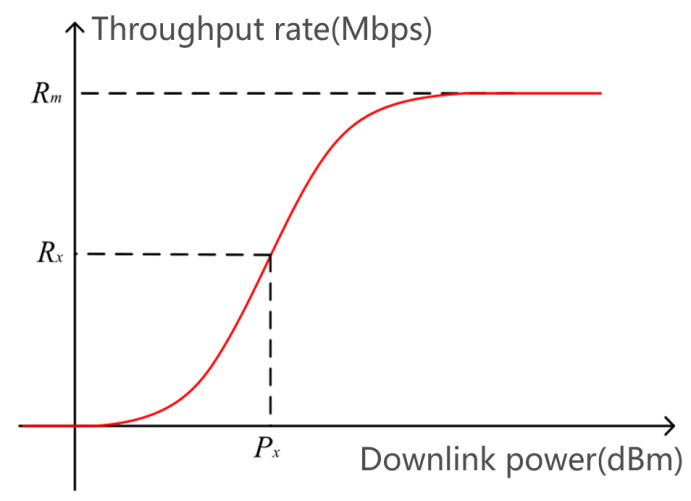
Typical throughput measurement result schematic.

Figure 11 presents a typical throughput measurement result, specifically the relationship curve between throughput and downlink power, where the downlink power P corresponding to the throughput R is taken as a benchmark. It can be observed that the throughput increases with the downlink power until it reaches the maximum throughput.

Following the analysis of the aforementioned preconditions, it is apparent that the magnitude of S-parameters significantly affects the MCL, which is defined as per 3GPP specifications. In the MCL’s definition formula, there exists a proportional relationship between the MCL and transmit power, provided that the terminal receiver sensitivity remains constant. Moreover, during testing in anechoic environments, the variability from external environments is relatively low, ensuring greater stability of the terminal receiver sensitivity. These conditions ensure that we can accurately assess signal transmission performance. As depicted in Figure 11, a positive proportional relationship exists between the downlink power and throughput until the maximum throughput is reached. This phenomenon further substantiates the proportional relationship between S-parameters and throughput, indirectly demonstrating the effectiveness and reliability of the electromagnetic compatibility radiation testing method.

Through research on this relationship, we systematically understand the principles of the electromagnetic radiation testing method. This foundation allowed us to conduct detailed simulation analyses focused on S-parameters in subsequent research. During the simulation process, we utilized CADFEKO simulation software to generate S-parameter data associated with electromagnetic radiation. These simulated data served as important references for evaluating communication system performance. Subsequently, we compared these simulation results to the dB values in relevant standards for comparative analysis. Through this comparison, we delved into the specific influences of S-parameters on system performance. This process provides an important reference for quality assessment and optimization of communication in practical applications.

## 4. Simulation Verification

**Figure 12:**
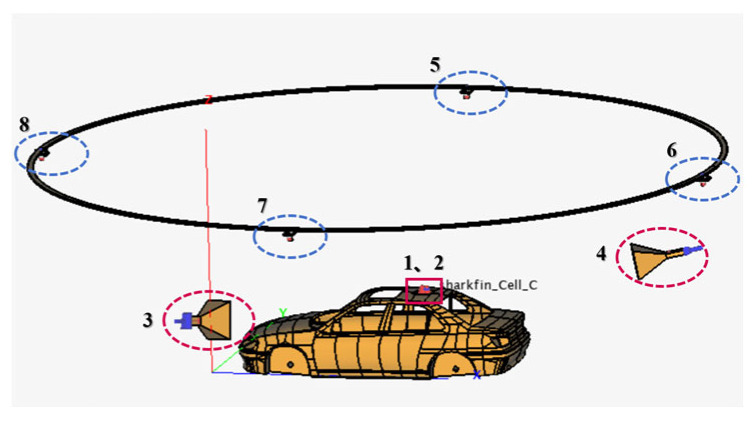
Arrangement of the transmission and receiving antennas within the model.

In establishing the three-dimensional electromagnetic model, we configured the vehicle-mounted receiving 4G and GNSS antennas as excitation ports 1 and 2 to accurately simulate their operating states within the vehicle. Meanwhile, to recreate the authentic communication environment, the simulated base station transmitting antenna was set as excitation ports 3 and 4. Additionally, to meet satellite communication requirements, the simulated satellite transmitting antenna was set as excitation ports 5, 6, 7, and 8. This port configuration scheme is shown in Figure 12, which comprehensively reflects the interactions among various antennas and their influence on signal propagation.

S-parameters, as coupling parameters between transmitting and receiving antennas, play a crucial role in our simulation process. To calculate the transmission coefficients from the transmitting antenna to the receiving antenna, we need to solve all relevant parameters listed in Table 1. It is particularly noteworthy that since the three-dimensional electromagnetic model we constructed involved signal transmission between multiple transmitting and receiving antennas, this simulation experiment was conducted within the framework of MIMO systems. Although this research focused on communication transmission performance between the base station and the vehicle’s 4G antenna, as well as between satellites and the vehicle’s global navigation satellite system antenna, it should be emphasized that these data do not equate to conclusions derived from SISO studies. In the MIMO environment, the simultaneous use of multiple antennas greatly enhances the capacity of the signal transmission, effectively improving the reliability and capacity of the communication system. Therefore, the results we obtained not only reflect the performance between base stations and antennas, but also encompass the effects of multi-node interactions in complex channel environments.

S-parameters, or scattering parameters, are critical metrics in microwave transmission, utilized to characterize the frequency domain properties of transmission channels. In passive devices, they represent interference and isolation, whereas in wireless transmission, they can denote the communication between antennas. The S-parameters consist of four fundamental reference quantities, namely S11, S12, S21, and S22, each encompassing distinct signal behaviors. S11 represents the reflection coefficient, characterizing the proportion of the signal reflected back from port 1. S12 denotes the reverse transmission coefficient, describing the signal transmission capability from port 2 to port 1. S21 signifies the transmission coefficient, illustrating the signal transmission capability from port 1 to port 2. S22, analogous to S11, represents the reflection coefficient, depicting the proportion of the signal reflected back from port 2. Given that this study focused on the signals transmitted from the base station and satellite to the vehicle-mounted antenna, the signal behavior of S21, which describes the signal transmission capability from port 1 to port 2, aligns well with the specific configuration of our designed transmitter and receiver. The reference values of the S-parameters required for our analysis are tabulated in Table 1. S23 describes the signal transmission capability from base station 3 to the vehicle-mounted 4G antenna, while S15 describes the signal transmission capability from satellite 5 to the vehicle-mounted GNSS antenna. The remaining S-parameters follow this pattern accordingly. During the design phase, we comprehensively considered channel characteristics, antenna configuration, and environmental factors to ensure that the listed parameters accurately represent the actual performance of the system. Furthermore, these data can be utilized to evaluate the overall performance of the communication system, including signal quality and stability, thereby providing a scientific basis for optimizing the design scheme.

**Figure 13:**
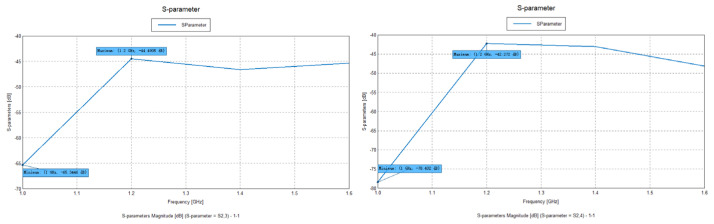

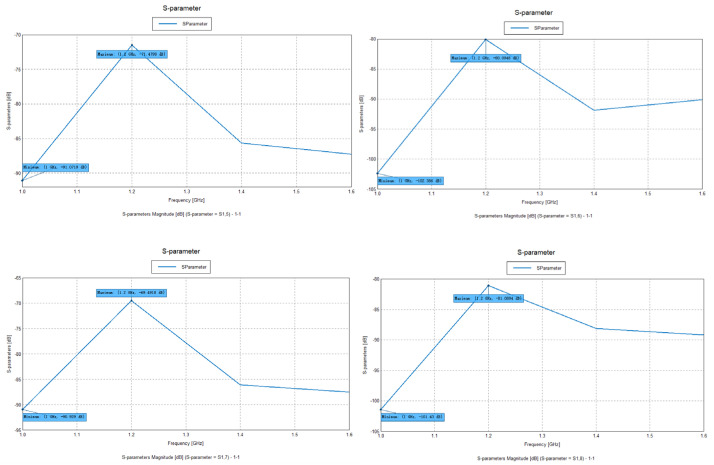
Simulation results of the S − parameters in the frequency range of 1 to 1.6 GHz.

After simulating and solving the three-dimensional electromagnetic model, we obtained all of the relevant parameters listed in Table 1, as shown in Figure 13. These parameters underwent in-depth statistical data analysis, ultimately leading to the summary of all S-parameters between the transmitting and receiving antennas, as shown in Table 2.

Specifically, Table 2 summarizes the transmission coefficients of the S-parameters at frequencies ranging from 1 to 1.6 GHz; these parameters are crucial indicators for assessing the coupling effect between the transmitting and receiving antennas, as well as the signal transmission performance. By analyzing these S-parameters, we can clearly understand the changes in signals during transmission and reception, thereby revealing the interactions among various antennas.

From Table 2, we can observe that the maximum absolute value of the S-parameters between the satellite and the vehicle’s GNSS antenna was 102.386, while the maximum absolute value of the S-parameters between the base station and the vehicle’s 4G antenna was 78.402. These values indicate that larger absolute S-parameter values signify a more severe coupling loss between the transmitting and receiving antennas. Such coupling loss directly affects signal transmission efficiency.

Table 3 illustrates that, according to the 3GPP standard specifications [11], the MCL is defined as a critical metric for evaluating the coverage range of wireless access technologies. Theoretically, the MCL can be interpreted as the maximum loss of transmitted power that the system can withstand while still functioning properly; it serves as the critical threshold for achieving normal data transmission. In other words, exceeding this loss level may render the system unable to maintain stable signal transmission.

From the data presented in Table 2 and Table 3, it is evident that although the S-parameter values obtained from the simulation are relatively high, they still fall short of meeting the MCL parameter specified in industry standards. This result indicates that there is further room for improvement in the current three-dimensional electromagnetic model. In other words, even if the S-parameter values are acceptable, there still exists potential for enhancement in performance and efficiency in practical applications.

At this point, the theoretical validation of the proposed method in this study has been completed. The next step involves replicating the research methodology in a fully anechoic chamber. This requires positioning the DUT, a robotic arm representing the base station, and a rail system representing the satellite in their respective locations. Subsequently, the throughput from the transmitting antenna to the vehicle-mounted antenna will be measured. By comparing the throughput data at the same transmission location with the S-parameters obtained from the simulation, the focus will be on assessing whether the fluctuations in the data nodes align between the two. For instance, if both the S-parameters and throughput data exhibit poor performance at the rear of the vehicle, this would demonstrate the feasibility of the proposed method.

Further analysis reveals that controlling the S-parameters can significantly influence the stability and quality of signal transmission. Therefore, targeted optimization of the design and implementation of the three-dimensional electromagnetic model can lead to improved performance. This can be achieved through adjustments such as modifying the antenna’s frequency band, selecting different materials, or optimizing the electromagnetic wave propagation environment. Such refinements will contribute to achieving superior performance outcomes. For instance, the simulation data indicate that different types of antennas exhibit varying effectiveness at different frequency points or bands. Hence, large-scale simulations can be employed to determine the optimal frequency points or bands for 4G or GNSS antennas; this represents one potential method for improvement. Additionally, selecting antennas with superior performance based on their characteristics or altering the installation positions of antennas on vehicles can serve as experimental conditions for refining the model. In summary, optimizing S-parameter values aims to further enhance the overall performance of wireless communication systems, ensuring their effectiveness and reliability in practical applications.

## 5. Conclusions

With the establishment of a closed-loop electromagnetic compatibility radiation testing method, we can utilize simulation software to optimize S-parameters, significantly improving the stability and performance of communication systems. This optimization not only aids in effectively reducing costs, but also ensures the repeatability and comparability of measurement results, laying a solid foundation for subsequent research and applications. Furthermore, this method allows for a more comprehensive and accurate assessment of the electromagnetic compatibility of connected vehicle communication antennas, enhancing their performance in complex environments, thereby further ensuring the safety and effectiveness of connected vehicles’ communications. This research method has already assisted a company in determining the optimal placement of antennas on vehicles in actual engineering applications.

The introduction of simulation testing enables us to accurately and reproducibly replicate the antenna propagation paths of real-world scenarios within an anechoic chamber, offering the advantage of providing consistent and comparable measurement results. However, establishing a simulation testing environment for measurements also presents certain limitations. First, the range of actual scenarios that simulation testing can represent is limited; it is primarily suitable for modeling classic environments and struggles to encompass all complex real-world conditions. Second, simulation testing typically does not account for the impact of external noise signals, which may lead to discrepancies between the results and the actual environment. Lastly, conducting measurements of the throughput between the transmitting and receiving antennas in practical experiments and comparing these with simulation data and the MCL would yield more comprehensive and refined results.

The proposed method in this study offers a simple and efficient approach to enhancing the efficiency and quality of wireless communication systems in connected vehicles. It particularly provides a powerful tool for antenna testing in commercial vehicles, significantly improving testing efficiency, reducing costs, and optimizing designs. However, the accuracy of the simulation results heavily depends on the precision of the models. A key focus for future research will be on developing electromagnetic field and channel models that more closely resemble real-world environments. Additionally, future work will refine practical testing by integrating actual test data and establishing mathematical models based on the relationship between throughput and S-parameters to further optimize the testing framework, thereby enhancing the practicality of the proposed method. It will also be essential to simulate or test other critical or specialized antennas on vehicles to prevent potential incompatibility issues.

Based on OTA performance simulation analyses across various fields, including 5G, millimeter-wave communication, the Internet of Things, and vehicular communication [12,13], combining the proposed method with OTA testing could unlock significant potential in applications such as V2X and 5G. This research not only contributes to the advancement of antenna testing techniques, but also supports the broader goal of enhancing the performance and reliability of connected vehicle communication systems. By improving testing efficiency and accuracy, this method has the potential to accelerate the development of next-generation automotive communication technologies, ultimately facilitating the realization of fully autonomous driving and seamless vehicle connectivity.

## Figures and Tables

**Table 1:** Statistical table of all the S-parameters that need to be solved.

	Base Station 3	Base Station 4	Satellite 5	Satellite 6	Satellite 7	Satellite 8
GNSS	/	/	S15	S16	S17	S18
4G	S23	S24	/	/	/	/

**Table 2:** S-parameters between the transmitting and receiving antennas.

	GNSS (dB)			4G (dB)		
3		/	/	S23	−44.491	−65.345
4		/	/	S24	−42.272	−78.402
5	S15	−71.4798	−91.0719	/	/	/
6	S16	−80.0948	−102.386	/	/	/
7	S17	−69.4918	−90.929	/	/	/
8	S18	−81.0894	−101.43	/	/	/

**Table 3:** MCL supported by different wireless access technologies.

S. No.	Radio Access Technology	MCL in dB
1	E-GPRS	~164 dB
2	LTE	~144 dB
3	LTE-M or Cat-M1	~160 dB
4	LTE-NB or NB-loT	~164 dB
5	SIGFOX	~162 dB
6	LORA	~157 dB

## Data Availability

Data are contained within the article.

## Abbreviations

The following abbreviations are used in this manuscript:

OTA	Over the air
DUT	Device under test
C-V2X	Cellular vehicle-to-everything
EMC	Electromagnetic compatibility
GNSS	Global navigation satellite system
NLOS	Non-line-of-sight
MIMO	Multiple-input and multiple-output
SISO	Single-input and single-output
V2V	Vehicle-to-vehicle
V2I	Vehicle-to-infrastructure
MCL	Maximum coupling loss
UE	User equipment
SINR	Signal-to-interference-plus-noise ratio
NF	Noise figure
3GPP	3rd Generation Partnership Project

## References

[1] Walkenhorst B.T., Pelland P., Leifert T., Berbeci M. A Survey of MIMO OTA Test Methodologies for Automotive Applications. Proceedings of the 2020 IEEE International Symposium on Antennas and Propagation and North American Radio Science Meeting, IEEECONF 2020—Proceedings.

[2] Pelland P., van Rensburg D.J., Berbeci M., Storjohann F.O., Griesche A., Busch J.P. (2020). Automotive OTA Measurement Techniques and Challenges. Proceedings of the 2020 Antenna Measurement Techniques Association Symposium, AMTA.

[3] Yang W. (2015). Research and Implementation of Multi-Probe MIMO OTA Testing System. Master’s Thesis.

[4] Shen P. (2020). Design and Implementation of MIMO Terminal Measurement System Based on Two-Step Radiation Method. Ph.D. Thesis.

[5] Zheng W. (2024). Upstream News. https://baijiahao.baidu.com.

[6] Xie Z., Zhang Y., Zhang Y., Li X. (2019). Spherical Near-Field and Far-Field Transformation Algorithm. J. Electromagn. Waves Appl..

[7] Altair + Feko Software Common Problems and Solutions 2022_V1(1)). https://2022.help.altair.com/2022.1.1/feko/messages/pdf/Altair_Feko_Errors_Warnings_Reference_Guide.pdf.

[8] 3GPP (2013). Technical Report (TR) 36.888: Study on Provision of Low-Cost Machine-Type Communications (MTC) User Equipment (UE) Based on LTE. https://www.3gpp.org.

[9] 3GPP (2022). Technical Specification (TS) 37.544: User Equipment (UE) Conformance Specification; Radio Transmission and Reception; Part 1: Range 1 Standalone. https://www.3gpp.org.

[10] 3GPP (2023). Technical Report (TR) 37.977: Study on Wide Area Network (WAN) Aspects of Industrial Internet of Things (IIoT). https://www.3gpp.org.

[11] 3GPP (2023). Technical Report (TR) 38.913: Study on Scenarios and Requirements for Next Generation Access Technologies. https://www.3gpp.org.

[12] Li Y., Wang L., Chen S. (2021). Simulation and Analysis of OTA Performance in Vehicular Communication Systems. IEEE Trans. Veh. Technol..

[13] Zhang J., Liu Y., Wang X. (2020). Simulation-Based Analysis of Over-the-Air Performance in 5G Networks. IEEE Commun. Mag..

